# The innovation bias: Implicit preferences for innovative and historical solutions over contemporary ones

**DOI:** 10.1016/j.isci.2025.112490

**Published:** 2025-04-18

**Authors:** Moritz Reis, Yeun Joon Kim, Roland Pfister, Wilfried Kunde

**Affiliations:** 1 Institute of Psychology, University of Wuerzburg, Roentgenring 11, 97070 Wuerzburg, Germany; 2 Cambridge Judge Business School, University of Cambridge, Trumpington Street, Cambridge CB2 1 AG, UK; 3 General Psychology, Trier University, Johanniterufer 15, 54290 Trier, Germany; 4 Institute for Cognitive & Affective Neuroscience (ICAN), Trier University, Universitätsring 15, 54296 Trier, Germany

**Keywords:** Social sciences

## Abstract

Innovative products and services promise to improve our lives in many ways. Novel, unfamiliar approaches, however, also come with increased uncertainty regarding their feasibility and quality. In four preregistered experiments, we investigated implicit biases toward such innovative approaches. We tracked hand movements while participants chose between options of different levels of innovativeness. Choices either compared historic versus contemporary options (past comparison; e.g., carriage vs. car) or they compared contemporary versus innovative options (future comparison; e.g., car vs. self-driving car). While for past comparisons, movement trajectories were systematically torn toward the more historic option, the opposite effect was observed for future comparisons. This pattern of results replicated across all four studies. People, thus, seem to implicitly favor innovative and historic approaches over established ones. We conclude that moderate incongruity from an established approach, either through innovation or through a return to the past, evokes particular interest and attraction toward non-standard alternatives.

## Highlights

• We use mouse tracking to capture implicit attitudes toward innovation
• People implicitly favor innovative and historic solutions over contemporary ones
• Deviations from the current standard thus exert an implicit attraction

## Graphical abstract

**Figure:**
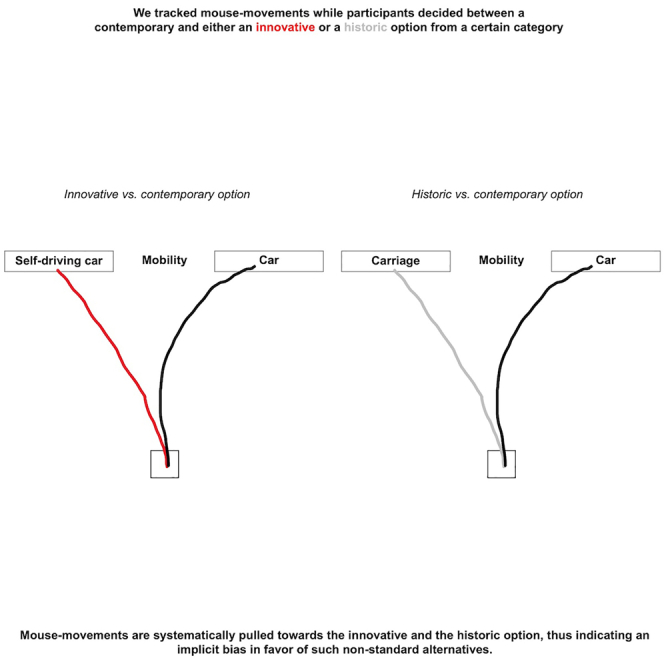

## Introduction

Human history houses countless examples of innovative solutions that have moved mankind forward.^1^ This includes historic achievements such as the printing press and electric light, as well as more recent innovations like computers or AI. Given this remarkable success story, one might assume that people strongly endorse innovative approaches, recognizing their potential to improve our world. However, in reality, innovations often face significant skepticism and rejection before establishing themselves in the market. For instance, at first, Thomas Edison’s light bulb was disregarded as a “conspicuous failure”^2^ by a leading scientist in the field, and the automobile was initially dismissed as a “passing fad”.^3^

In line with these inconsistent real-world observations, academic research has also yielded perplexing findings. On the one hand, some researchers have suggested that consumers tend to prefer innovative products over traditional ones.^4^ This line of research argues that innovative products spark customers’ curiosity, leading them to become eager to learn more about their potential benefits.^5^ Such a preference for innovation was particularly evident under two conditions: When the expectation of failure in choosing an innovative product did not entail dramatic consequences and when a given innovative product only moderately deviated from the previous standard in its product category.^6^^,^^7^^,^^8^

On the other hand, another line of research argues that individuals exhibit a bias against innovation. Innovative products and approaches can not only provide substantial benefits but also stand out due to their novelty and unusualness.^9^ Novel, previously unknown approaches, in turn, carry increased uncertainty regarding their feasibility and might threaten one's sense of control.^10^ In other words, while an established product has repeatedly demonstrated its benefits, an innovative product, characterized by high novelty, typically lacks the same level of demonstrated reliability.^11^

To assess preferences for or against innovation, previous studies, however, utilized explicit measures (ratings). Such measures are subject to several biases like social desirability and demand effects.^12^^,^^13^ As innovation is seen as highly socially desirable, these limitations are of particular relevance here.

The goal of this research, thus, is to gain a better understanding of how people perceive innovative solutions by applying a less obtrusive, implicit measure. This necessity receives further support from research on creativity, a concept closely related to innovation.^14^ In particular, it has been shown that despite their positive connotation, people are implicitly biased against creative ideas, especially under conditions of increased uncertainty.^15^ This implicit bias is particularly evident in so-called action-dynamics paradigms that track cognitive pulls and pushes via movement trajectories: when choosing between traditional and creative ideas, there is an implicit bias toward the more traditional option, even when selecting the creative one eventually.^16^^,^^17^^,^^18^^,^^19^ Accordingly, human behavior appears to lean toward conventional, more familiar ideas.

Whether there is a similar implicit bias against innovation, however, is yet to be explored. Even though the conceptual overlap of creativity and innovation might suggest a similar pattern, both concepts also differ in crucial aspects. Given that perceived uncertainty is the underlying mechanism which drives biases against creativity,^15^ the eventual implementation of a creative idea, i.e., innovation, might be sufficient to counteract this uncertainty.

### The present research

To unravel implicit attitudes toward innovation, we developed an action-dynamics paradigm in which participants were tasked with choosing between two options within a specific product category (e.g., mobility). Crucially, both options differed in their degree of innovativeness. That is, one option was a contemporary instance of this category (e.g., car), while the other option was either a less innovative, historic representative (e.g., carriage) or a more innovative, future-oriented representative (e.g., self-driving car). The key feature of our paradigm is to capture participants’ behavioral inclination toward one of the two options. Participants made their choice between the two options by moving the mouse cursor from the bottom center of the screen to the corresponding target areas in the upper right and upper left corners of the computer screen. Based on models of dynamic decision-making in cognitive science,^20^ analyzing these movement trajectories allows to explore the moment-to-moment changes of participants’ response selection. Thereby, this action-dynamics approach reveals the hidden decision-making process, which underlies observable behavior, and offers a measure of implicit attraction toward a response option.^21^^,^^22^^,^^23^ That is, if a movement going for one option is slower and more curved toward the opposing option than vice versa, this may indicate an implicit bias toward the non-chosen option. While we here introduce this approach to innovation research, comparable paradigms have been validated in other research fields. For instance, violating a simple stimulus-response rule was found to yield a bias toward the rule-compliant alternative^24^ and, similarly, dishonest responses to yes/no questions were shown to be biased toward the honest response option.^25^

Comparing spatial and temporal characteristics of the performed mouse-movements toward more or less innovative options thus allowed us to pinpoint an implicit bias either toward or against innovation. Testing the currently established approach against both a previous one and a future innovation within this category, further enabled us to explore how implicit attitudes toward innovation change over time. That is, options may not only deviate from the current standard in terms of increased novelty but also in terms of increased old-fashionedness. Both kinds of deviations should be less familiar to the consumer than contemporary approaches and may thus be linked to enhanced uncertainty (for an overview on different kinds of atypicality in organizational and market settings, see Cutolo and Ferriani).^26^ At the same time, a rather historic option has already (at some point in time) successfully established itself on the market. Even though this establishment may have happened some time ago, it may be perceived as a proof of the option’s reliability and therefore reduce its uncertainty. In particular radical innovations can even change the perception of the product category itself, making the innovation the new standard in the respective field.^27^

We implemented this setup in four preregistered online experiments (*N* = 272 adults across different nationalities). Between experiments, we varied several aspects of our setup, such as whether the respective product category was explicitly mentioned or not (Experiment 1 vs. Experiment 2), the wording of the labels (“traditional” and “innovative” vs. “less innovative” and “more innovative”; Experiment 3) and the presentation format of the response options (images vs. text; Experiment 4). Table 1 provides an overview of the methodological differences between all experiments.

**Table 1:** Overview of all experiments

	Product category	Stimulus labels	Stimulus format
Experiment 1	absent	Traditional vs. Innovative	Images
Experiment 2	present	Traditional vs. Innovative	Images
Experiment 3	present	Less vs. More innovative	Images
Experiment 4	present	Less vs. More innovative	Text

## Results

**Figure 1:**
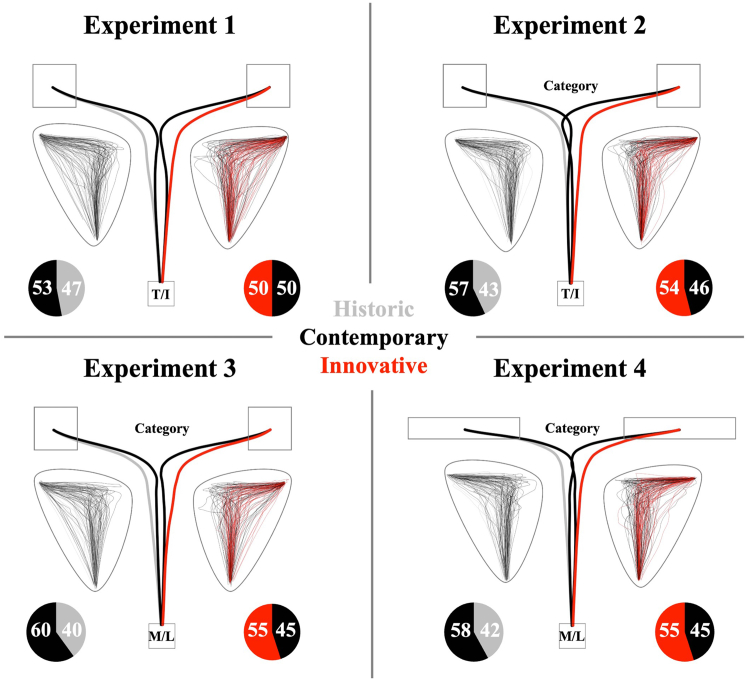
Setup and main results of Experiment 1–4 Bold lines show time-normalized movement trajectories when participants either selected a historic (gray line), a contemporary (black line) or an innovative option (red line) out of a certain category. Left going trajectories indicate the comparison between the historic and the contemporary option, while right going trajectories indicate the comparison between the contemporary and the innovative option. Thin lines represent average trajectories of single participants for the respective conditions. Pie charts show the share of historic, contemporary, and innovative choices.

Figure 1 presents average trajectories and choice frequencies for all conditions within each experiment. Tables S2–S5 in the supplement show means and standard deviations of IT, MT and AUC for each combination of comparison type and targeted innovativeness across experiments.

### Experiment 1

When prompted to choose between the historic and the contemporary approach, there was a descriptive, however statistically not significant, preference for the more innovative option, *t*(67) = 1.93, *p* = 0.058, *d*_*z*_ = 0.23 95%-CI [-0.01, 0.47]. For trials in which the contemporary option was compared to the innovative one (future comparison), choice frequencies did not indicate any difference between both response options, |*t*| < 1. ITs did not differ between comparison type, *F*(1, 60) = 1.18, *p* = 0.282, η_p_^2^ = 0.02, or targeted innovativeness, *F* < 1. Also, there was no interaction of both factors, *F* < 1. MTs were significantly higher for future comparisons relative to past comparisons, *F*(1, 60) = 10.60, *p* = 0.002, η_p_^2^ = 0.15, and significantly higher when aiming at the more traditional rather than the more innovative objects, *F*(1, 60) = 22.09, *p* < 0.001, η_p_^2^ = 0.27. Furthermore, comparison type and innovativeness interacted significantly, *F*(1, 60) = 29.70, *p* < 0.001, η_p_^2^ = 0.33. That is, for past comparison trials, MTs did not differ significantly between both innovativeness conditions, *t*(60) = 1.08, *p* = 0.286, *d*_*z*_ = 0.14 95%-CI [-0.11, 0.39], but for future comparison trials, MTs were significantly higher when participants aimed for the more traditional than when aiming for the more innovative object, *t*(60) = 6.27, *p* < 0.001, *d*_*z*_ = 0.80 95%-CI [0.51, 1.09]. AUCs did not show main effects of comparison type, *F*(1, 60) = 2.35, *p* = 0.130, η_p_^2^ = 0.04, or innovativeness condition, *F*(1, 60) = 1.83, *p* = 0.181, η_p_^2^ = 0.03, but there was a strong interaction of both factors, *F*(1, 60) = 62.13, *p* < 0.001, η_p_^2^ = 0.51. For past comparisons, AUCs were significantly larger when aiming for the more innovative, contemporary object, *t*(60) = 4.47, *p* < 0.001, *d*_*z*_ = 0.57 95%-CI [0.30, 0.84], but AUCs were significantly smaller when aiming for the more innovative object for future comparison trials, *t*(60) = 7.31, *p* < 0.001, *d*_*z*_ = 0.94 95%-CI [0.63, 1.23]. In other words, trajectories were torn toward the historic object in past comparisons, whereas they were torn toward the innovative object in future comparisons. Please note that this effect (as well as all other significant effects reported in the following) remains stable if calculating a mixed-effect analysis controlling for the random effect of test items in the different categories.

### Experiment 2

For both comparison types, participants were more likely to choose more innovative than traditional options (past comparison: *t*(67) = 3.27, *p* = 0.002, *d*_*z*_ = 0.40 95%-CI [0.15, 0.64]; future comparison: *t*(67) = 2.08, *p* = 0.041, *d*_*z*_ = 0.25 95%-CI [0.01, 0.49]). For ITs, there was neither an effect of comparison type or innovativeness condition, nor an interaction of both factors, *F*s < 1. The results for MTs paralleled our findings from Experiment 1 (comparison type: *F*(1, 56) = 8.23, *p* = 0.006, η_p_^2^ = 0.13, innovativeness: *F*(1, 56) = 13.55, *p* = 0.001, η_p_^2^ = 0.19, interaction of comparison type and innovativeness: *F*(1, 56) = 20.16, *p* < 0.001, η_p_^2^ = 0.26). In line with the results of the first experiment, MTs were not affected by innovativeness for past comparison trials, |*t*| < 1, but for future comparisons they were significantly higher for traditional compared to innovative trials, *t*(56) = 5.42, *p* < 0.001, *d*_*z*_ = 0.72 95%-CI [0.42, 1.01]. AUCs were descriptively, however not statistically significantly larger for future compared to past comparison trials, *F*(1, 56) = 3.69, *p* = 0.060, η_p_^2^ = 0.06. As in Experiment 1, there was a main effect of innovativeness, *F*(1, 56) = 11.32, *p* = 0.001, η_p_^2^ = 0.17, as well as an interaction of comparison type and innovativeness, *F*(1, 56) = 62.14, *p* < 0.001, η_p_^2^ = 0.53. For past comparisons, AUCs were significantly larger for innovative choices, *t*(56) = 3.58, *p* = 0.001, *d*_*z*_ = 0.47 95%-CI [0.20, 0.75]. In contrast, for future comparisons, AUCs were significantly larger for traditional choices, *t*(56) = 7.68, *p* < 0.001, *d*_*z*_ = 1.02 95%-CI [0.69, 1.34].

### Experiment 3

Similar to Experiment 2, choice frequencies indicated a strong preference for innovative over traditional options, irrespective of comparison type (past comparison: *t*(67) = 4.89, *p* < 0.001, *d*_*z*_ = 0.59 95%-CI [0.33, 0.85]; future comparison: *t*(67) = 3.13, *p* = 0.003, *d*_*z*_ = 0.38 95%-CI [0.13, 0.62]). For ITs, there were no significant main effects or interactions, *F*s < 1. MTs were significantly higher for future comparisons relative to past comparisons, *F*(1, 52) = 12.97, *p* = 0.001, η_p_^2^ = 0.20, but there was no main effect of innovativeness, *F* < 1. Both factors interacted significantly, *F*(1, 52) = 30.43, *p* < 0.001, η_p_^2^ = 0.37. For past comparisons, MTs were significantly higher for more innovative trials, *t*(52) = 2.64, *p* = 0.011, *d*_*z*_ = 0.36 95%-CI [0.08, 0.64], while the opposite effect emerged for future comparisons, *t*(52) = 3.18, *p* = 0.002, *d*_*z*_ = 0.44 95%-CI [0.15, 0.72]. We found significantly larger AUCs for future comparisons relative to past comparisons, *F*(1, 52) = 11.85, *p* = 0.001, η_p_^2^ = 0.19. There was no main effect of innovativeness on AUCs, *F*(1, 52) = 1.74, *p* = 0.193, η_p_^2^ = 0.03, but innovativeness and comparison type interacted significantly, *F*(1, 52) = 61.56, *p* < 0.001, η_p_^2^ = 0.54. In line with the results of Experiment 1 and 2, AUCs were significantly larger for innovative selections in past comparison trials, *t*(52) = 4.05, *p* < 0.001, *d*_*z*_ = 0.56 95%-CI [0.26, 0.84], but significantly larger for traditional selections in future comparison trials, *t*(52) = 5.42, *p* < 0.001, *d*_*z*_ = 0.74 95%-CI [0.44, 1.05].

### Experiment 4

The analysis of choice frequencies, paralleled our findings from Experiment 2 and 3 (past comparison: *t*(67) = 4.19, *p* < 0.001, *d*_*z*_ = 0.51 95%-CI [0.25, 0.76]; future comparison: *t*(67) = 2.75, *p* = 0.008, *d*_*z*_ = 0.33 95%-CI [0.09, 0.58]). There was neither an effect of comparison type or innovativeness on ITs, nor did both factors interact significantly, *F*s < 1. MTs were not affected by comparison type, *F* < 1, but were significantly higher for more traditional compared to more innovative choices, *F*(1, 54) = 11.18, *p* = 0.002, η_p_^2^ = 0.17. Both factors interacted significantly, *F*(1, 54) = 31.34, *p* < 0.001, η_p_^2^ = 0.37. That is, for past comparisons MTs did not differ significantly between both innovativeness conditions, |*t*| < 1, but for future comparisons MTs were significantly higher for traditional compared to innovative trials, *t*(54) = 6.75, *p* < 0.001, *d*_*z*_ = 0.91 95%-CI [0.59, 1.22]. Similarly, AUCs did not differ between future and past comparison trials, *F* < 1, were significantly larger for traditional trials, *F*(1, 54) = 4.78, *p* = 0.033, η_p_^2^ = 0.08, and both factors interacted significantly, *F*(1, 54) = 69.13, *p* < 0.001, η_p_^2^ = 0.56. For past comparisons, AUCs were significantly larger for innovative compared to traditional trials, *t*(54) = 4.15, *p* < 0.001, *d*_*z*_ = 0.56 95%-CI [0.27, 0.84], while the opposite effect emerged for future comparisons, *t*(54) = 6.80, *p* < 0.001, *d*_*z*_ = 0.92 95%-CI [0.60, 1.23].

## Discussion

Are individuals implicitly biased toward or against innovation? We explored this question in four preregistered experiments by tracking hand movements while participants chose between more and less innovative options out of certain categories. In half of the trials, they had to choose between a rather historic and the contemporary option (e.g., carriage vs. car). In the other half of the trials, the contemporary option was compared to a recent innovation within this field (e.g., self-driving car). Between our four experiments, we varied certain aspects of the experimental setup, like labeling and presentation format of the response options and whether the respective product category was explicitly mentioned or not.

Crucially, when testing the contemporary against a future solution, corresponding movement trajectories indicated a lasting bias in favor of the more innovative option. This finding replicated consistently across all four studies but stands in strong contrast to observations in the field of creativity, which documented a strong, implicit bias against creative ideas.^15^^,^^17^ These diverging results indicate a crucial difference between innovation and creativity. In general, both concepts are characterized by leaving a beaten path and taking a step into the unknown.^28^ However, while creative ideas might be particularly abstract and ambiguous, an innovative solution has, at least to some extent, already proven its practicability. That is, as soon as creative ideas are practically implemented and therefore become more tangible, a consistent bias against creative ideas may turn into a consistent bias toward innovative solutions. In this case, remaining uncertainty regarding the innovation may be overridden by the potential benefits the novel approach offers.^5^ This assumption is in line with recent evidence, suggesting that implicit biases against creative ideas are markedly reduced for own compared to other people's ideas.^18^ For such self-generated ideas, confidence in the practicability of the original approach may be more pronounced than for externally provided options, thus reducing the inherent uncertainty of creative ideas. Along the same lines, reducing uncertainty by establishing clear rules of conduct has been found to promote creativity in organizational settings.^29^

In contrast, when participants had to choose between the contemporary and an old-fashioned approach, we found a significant pull toward the more historic option. As both options were established approaches within the respective domain at different points in time, differences in perceived uncertainty seem unlikely to drive this effect. What, however, unifies highly innovative and outdated options is that they both are less familiar than current options. Thus, both innovative and outdated approaches might induce interest and curiosity in the observer. Along these lines, research on vintage consumption found that products that are viewed as “of the past” are seen as scarcer, which in turn enhances their subjective value for the customer.^30^ Such a return to the past can thus help to express one’s uniqueness and authenticity.^31^^,^^32^ Moreover, by connecting past, present, and future, vintage (i.e., historic) items can create a sense of intertemporal interconnection, which bolsters people against meaning threats.^33^ Summarized, it thus seems that any deviation from the current standard, even if it is a return to a previous standard, exerts an implicit attraction.

From a practical perspective on innovation management our findings indicate that innovation needs a long-term perspective to strive. Innovations in a very early stage (i.e., creative ideas) may be primarily characterized by enhanced uncertainty, which often results in rejection by investors and superiors.^34^ During the implementation process this uncertainty, however, seems to decline and initial skepticism may change into strong attraction.^35^ Innovators, thus, should brace for initial rejection while countering this rejection by emphasizing the practicability of their early-stage ideas and by aiming to make their ideas more concrete as soon as possible. Future research could apply the present action-dynamics approach to investigate more closely how implicit attitudes toward an innovation change throughout the implementation process (e.g., when a first prototype is available). By doing so, it would be possible to identify the most crucial milestones during the innovation journey. Moreover, it often may be advisable to limit the degree of novelty and focus on incremental innovations as these should be perceived as less uncertain. In line with this assumption, previous research found that in most contexts, people only prefer innovative over traditional solutions provided that the degree of novelty is limited. As historic and innovative options within our study were evaluated as moderately typical for the corresponding category (see Table S1), this condition should be met within our setup. This so-called *moderate incongruency effect*^7^^,^^8^ can be found in various contexts, including advertisement, taste and movie preferences. ^36^^,^^37^^,^^38^ Thus, a certain level of novelty may induce customers’ curiosity and create positive feelings. Yet, too much novelty seems to be experienced as aversive, because it violates customers’ expectations of a new product. Due to this curvilinear relation between novelty and liking, innovators can often benefit from deliberately reducing the novelty of their work. Paradoxically, this novelty reduction can even lead to a more innovative result in the long term, provided that the novelty reduction is either restricted to certain aspects of the product or to a certain stage of the innovation process.^39^ An exciting avenue for future research may be to experimentally manipulate the degree of novelty of the provided response options. If the present bias toward both kinds of non-standard options (innovative and historic) declines with increasing atypicality, this would be strong evidence in favor of the moderate incongruency assumption.

Lastly, our findings suggest the possibility of a paradoxical co-existence between future- and past-orientations, which may be the main driver for innovation. A society that focuses solely on future-oriented innovation risks disapproving of or undervaluing its past and present. In contrast, a society that only appreciates the past may struggle to achieve innovation. In reality, however, we frequently revisit and value our past while simultaneously pursuing for future innovations. This co-existence, although seemingly paradoxical, may reflect a fundamental human nature that underlies societal progress. Indeed, creativity scholars have argued that embracing paradox can be a powerful driver for innovation.^40^^,^^41^^,^^42^ Future research may investigate whether the paradoxical coexistence of past- and future-orientation can indeed stimulate innovation.

### Conclusion

In four preregistered experiments, we found that people implicitly prefer innovative and historic solutions over currently established ones. We conclude that deviations from the standard approach can be particularly attractive, regardless of whether it is a deviation toward the past (historic) or toward the future (innovation).

### Limitations of the study

As a limitation, it should be noted that the presented instances of each category differed in several aspects next to innovativeness (e.g., a bitcoin is a virtual object while a credit card is a physical object). To account for this issue, we calculated additional mixed-effect analyses. Crucially, all presented findings remain stable when controlling for the random effect of item category. As such, it seems highly likely that the reported differences are indeed due to varying degrees of innovativeness instead of other characteristics of the used stimuli. Along these lines, it is important to mention that there are many kinds of innovation (e.g., regarding the function or the design of a product). While in some contexts, people may for instance prefer a rather historic design (e.g., vintage cars), they may not desire the reduced functionality of a historic option (e.g., reduced speed and acceleration). Furthermore, while people may prefer a more historic option for a special occasion (e.g., traveling with a horse-drawn sleigh as part of a guided city tour), they would not prefer such an option for their daily travels. Future research should more closely dissociate the kind of innovation (e.g., form or function) that could be done by manipulating characteristics of the provided stimuli and also put more emphasis on the decision-making context (e.g., one-off or regular decision).

Moreover, the present studies aimed to assess individuals’ preferences for or against innovation in a highly controlled environment. While this approach provides high internal validity of our findings, it comes at the cost of reduced external validity. Thus, future research should aim to replicate our findings in a more ecological setup. For instance, participants’ decisions could be linked to real-world consequences (e.g., conducting a task with the chosen approach) and the provided options could be adapted so that they more closely represent real-world decisions. Furthermore, we conducted several exploratory analyses regarding interindividual differences of our findings (e.g., regarding demographic information, personality factors and political orientation; see supplement for details). While neither of these analyses indicated a significant relation of the results in the main experiments and participant-related factors, it should be noted that the present studies were not appropriately powered for such correlational investigations. Future research with larger sample sizes is required to provide a clearer picture of interindividual differences regarding the present findings. This also regards to the influence of gender on the results, which has not been investigated in the present study. Along these lines, also potential effects of additional variables like individuals’ cultural and socio-economic background would be of great interest.

## Resource availability

### Lead contact

Requests for further information and resources should be directed to Moritz Reis (moritz.reis@uni-wuerzburg.de).

### Materials availability

All materials have been deposited at the Open Science Framework and are publicly available at https://osf.io/25eb7/ as of the date of the publication.

### Data and code availability

• Data of all experiments has been deposited at the Open Science Framework and is publicly available at https://osf.io/25eb7/ as of the date of the publication.
• All original code has been deposited at the Open Science Framework and is publicly available at https://osf.io/25eb7/ as of the date of the publication.
• Any additional information required to reanalyze the data reported in this paper is available from the lead contact upon request.

## Acknowledgments

This research was supported by the Department of Psychology III of the University of Wuerzburg. Roland Pfister is funded by the Heisenberg Programme of the German Research Foundation (PF853/10-1).

## Author contributions

M.R. served as lead for conceptualization, methodology, formal analysis, visualization, and writing–original draft. W.K. served as lead for funding acquisition. W.K. and R.P. contributed equally to conceptualization and methodology. Y.J.K., W.K., and R.P. contributed equally to writing–review and editing and supervision.

## Declaration of interests

The authors declare no competing interests.

## STAR★Methods

### Key resources table

**Table:** 

REAGENT or RESOURCE	SOURCE	IDENTIFIER
**Deposited data**		

Data for all experiments	This paper	https://osf.io/25eb7/
Analysis code for all experiments	This paper	https://osf.io/25eb7/

**Software and algorithms**		

R	R Core Team^43^	https://www.r-project.org
mousetrap	Kieslich & Henninger^44^	https://doi.org/10.3758/s13428-017-0900-z
lab.js	Henninger et al.^45^	https://doi.org/10.3758/s13428-019-01283-5

**Other**		

Preregistration for study 1	This paper	https://osf.io/6fxuc/
Preregistration for study 2	This paper	https://osf.io/nvs82/
Preregistration for study 3	This paper	https://osf.io/bhn43/
Preregistration for study 4	This paper	https://osf.io/8xhvw/

### Experimental model and study participant details

#### Experiment 1

For the first experiment, we recruited 68 participants, out of which 61 participants were included in the final sample as per our preregistered exclusion criterion (i.e., less than ten valid trials per innovativeness condition after exclusions; 19 females, 41 males, 1 non-binary; age: *M* = 31.9, *SD* = 13.4 years). This sample size provided a statistical power of 1-β = 80% to detect effect sizes of *d*_*z*_ ≥ 0.36. Participants indicated 21 different nationalities (most common: the UK, *n* = 10, and Poland, *n* = 10). For all experiments, we applied a 2x2 within-subjects design (chosen target innovativeness: relatively high vs. low; type of comparison item: past vs. future), so all participants went through all experimental conditions in randomized order. The experimental procedure for each study was approved by the local ethics committee and all experiments were conducted in accordance with the ethical standards of the American Psychological Association.

#### Experiment 2

For the second study, we collected a new sample of 68 participants, out of which 57 individuals met our preregistered inclusion criterion (21 females, 35 males, 1 non-binary; age: *M* = 28.6, *SD* = 7.5 years; 1-β = 80% for *d*_*z*_ ≥ 0.37). Again, we recruited a diverse, international sample (23 different nationalities), with Poland (*n* = 12) and Portugal (*n* = 9) as the most common responses.

#### Experiment 3

Similar to Experiment 1-2, we collected data of 68 participants for Experiment 3. The data of 15 participants had to be excluded due to our preregistered exclusion criterion, leading to a final sample size of 53 participants (28 females, 25 males; age: *M* = 28.6, *SD* = 6.7 years; 1-β = 80% for *d*_*z*_ ≥ 0.39). Overall, 20 different nationalities were indicated, and South Africa (*n* = 12) and the UK (*n* = 8) were mentioned most frequently.

#### Experiment 4

For the final experiment, a new sample of 68 participants was recruited. Again, participants with less than ten valid trials per innovativeness condition were excluded (*n* = 13). Thus, the final sample consisted of 55 participants (27 females, 27 males, 1 non-binary; age: *M* = 29.4, *SD* = 10.6 years; 1-β = 80% for *d*_*z*_ ≥ 0.38; 18 different nationalities, most common nationalities: South Africa, *n* = 11, and the UK, *n* = 9).

### Method details

#### Experimental methodology: Overview

All studies followed a similar experimental procedure with only slight differences as outlined in Table 1. We recruited our samples via *Prolific*.

#### Stimuli and procedure

We selected ten different product categories, for which we generated three objects each (historic, contemporary, innovative). We conducted a prestudy (*N* = 60), to ensure that these objects differed in their degree of innovativeness as we expected (see Data S1 in the supplement for details).

The experimental setup was programmed with *lab.js*^45^ and the mouse-tracking plugin *mousetrap*.^44^ We used a reference screen size of 800 x 600 px, but the study was scaled depending on each participant′s individual display resolution. After providing informed consent, participants received detailed instructions on their task. At the start of each trial, participants indicated their intention for the upcoming trial (selecting a traditional or an innovative object; see Figure S1). To do so, they had to click on one out of two boxes. These were labelled “Traditional object” and “Innovative object” for Experiments 1-2, and “Less innovative object” and “More innovative object” for Experiments 3-4. The position of each box was determined randomly but constant for each participant during the entire experiment. We queried this intention before each trial to ensure that participants process the presented options and to allow us to differentiate deliberate choices and errors. To center the mouse cursor position, we next presented a small black square in the middle of the screen, on which participants had to click to proceed. The next screen featured three areas: Two target areas (Experiment 1-3: 100 px x 100 px; Experiment 4: 250 px x 50 px) in the upper left and upper right side of the screen and a home area (60 px x 60 px) in the bottom center. Within the home area we presented a reminder on participants’ intention for this trial (Experiment 1-2: “I” for innovative object, “T” for traditional object; Experiment 3-4: “L” for less innovative object, “M” for more innovative object). One target area showed the traditional object and the second target area showed the innovative object, with randomized mapping for each trial. Objects appeared after participants had clicked on the home area and they were presented as images in Experiments 1-3, whereas they were presented as text for Experiment 4. For Experiments 2-4, we additionally showed the respective product category in written form between both response options. Participants were instructed to select the chosen object as fast as possible by mouse click. We measured the required time to leave the home area (Initiation Time, IT) and the time from leaving the home area until approaching the center of one of both target areas (Movement Time, MT), defined as entering a circle of 20 px radius around the target center. To analyse mouse cursor trajectories, we sampled corresponding x- and y-coordinates throughout the movement. We did not present feedback and the next trial commenced directly after clicking on one of both target areas. Overall, there were five blocks with 40 trials each. At the end of the study, we collected additional measures (e.g., demographics) for exploratory analyses (see supplement for details).

### Quantification and statistical analysis

#### Sample size estimation

Previous studies, using a mouse-tracking paradigm to study creative idea selection, found small effect sizes of *d*_*z*_ = 0.40.^17^ We aimed for a power of 1-β = 80% to detect such an effect, resulting in a minimum sample size of 51 participants (α = .05, two-tailed testing, computed in R, version 4.1.1, via the *power.t*.*test* function of the *statistics* package).^43^ As we preregistered to only include participants with at least ten valid trials per condition (i.e., after exclusions),^46^ we increased our sample size by 30%. Accordingly, for each experiment, we collected data of 68 participants.

#### Analysis plan

For each study, similar exclusion criteria and statistical analyses were applied. To analyze mouse cursor movements, we preprocessed the trajectory data with custom R scripts (the detailed analysis algorithm can be found in the project repository on the Open Science Framework; https://osf.io/25eb7/; for more information on how to design mouse-tracking experiments and analyze corresponding data see Kieslich et al.,^47^ Pfister et al.^48^ and Wirth et al.^46^). Due to the online setup (and therefore varying screen sizes) we first scaled all trajectories to a uniform display resolution. For all scaled trajectories the distance from each target area’s center and the home area was 100 x-units (xu) on the x-axis and 200 xu on the y-axis. Further, to allow a comparison of all movements, left-going trajectories were mirrored at the vertical midline, so all trajectories went to the right target area. Then, trajectories were time-normalized to 101 points from movement onset to reaching the target area by linear interpolation. In addition, we appended the last coordinate of the movement after time normalization, to compensate for varying dwell times within the target area. The signed area between these scaled coordinates and a straight line from start- to endpoint of the mouse-cursor movement was determined as the area under the curve (AUC, in xu^2^).

Choice frequencies were calculated based on all available trials. As preregistered, for analyses of performance data (IT, MT, AUC), we then excluded error trials (i.e., opting for the more innovative option when having indicated to go for the less innovative one and vice versa) and extreme outliers (IT > 3,000 ms, MT > 5,000 ms, AUC > 30,000 xu^2^, and AUC < -20,000 xu^2^; cut-off values are based on prior investigations using a comparable study design^17^). After applying these filters, we further excluded trials which deviated more than 2.5 *SD*s from the corresponding cell mean and trials with less than three data points, as here no meaningful AUC can be calculated.

For choice frequencies, we conducted two separate two-tailed *t*-tests comparing more and less innovative responses for each comparison type. For each performance measure (IT, MT, AUC), we then computed separate repeated-measures analyses of variance (rmANOVAs), using the chosen target innovativeness (relatively high or low) and the type of comparison item (past or future) as within-subject factors. Significant interaction effects were followed by two-tailed *t*-tests comparing the respective measure between high and low innovative trials for each comparison type.

### Additional resources

All experiments were preregistered (Exp 1: https://osf.io/6fxuc/; Exp 2: https://osf.io/nvs82/; Exp 3: https://osf.io/bhn43/; Exp 4: https://osf.io/8xhvw/).
