# Supplementary material for: The innovation bias: Implicit preferences for innovative and historical solutions over contemporary ones
Source: iScience. 2025 Apr 18;28(5):112490. doi: 10.1016/j.isci.2025.112490 (PMC12124592; doi:10.1016/j.isci.2025.112490)
Supplement: Document S1. Figure S1, Tables S1–S5, and Data S1 [file mmc1.pdf]

iScience, Volume *28*

Supplemental information

The innovation bias: Implicit preferences for innovative and historical solutions over contemporary ones

Moritz Reis, Yeun Joon Kim, Roland Pfister, and Wilfried Kunde

Content

- Figure S1: Trial procedure
- Table S1: Categories and corresponding items for all experiments
- Table S2-S5: Descriptive statistics for all experiments
- Data S1: Prestudy
- Supplementary results: Interindividual differences

Figure S1. Trial procedure, related to STAR Methods

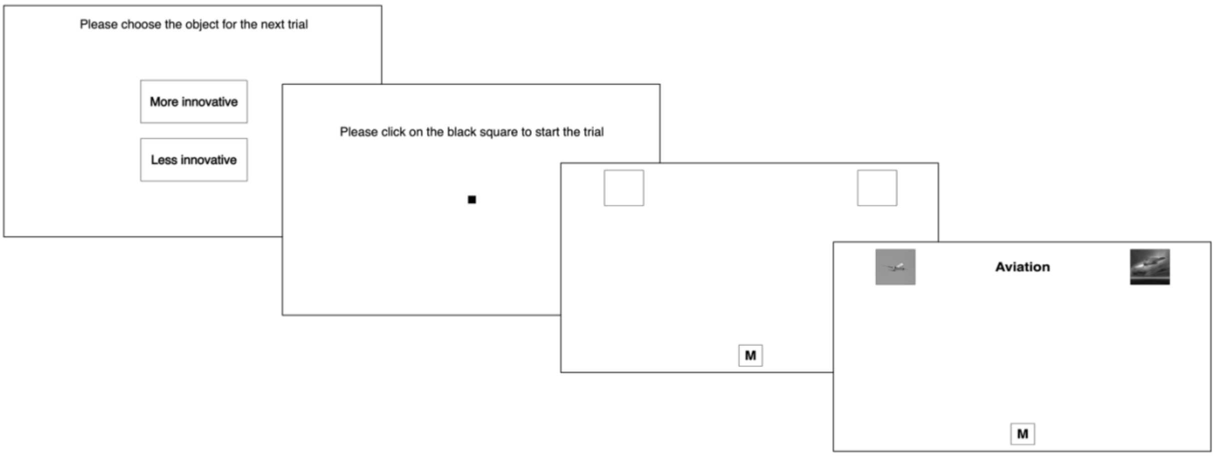

Fig. S1. Trial procedure. Participants indicated their intention for the upcoming trial, and we centered the mouse cursor position. After clicking on the home are, the response options and the corresponding category appeared. Participants now had to select the chosen option as fast as possible. For slight differences of the exact trial procedure between the experiments see Table 1.

Table S1. Categories and corresponding items for all experiments, related to STAR Methods

Category	Historic	Contemporary	Innovative
Mobility	Carriage	Car	Self-driving car
Aviation	Glider	Plane	Spaceship
Visual aid	Monocle	Glasses	VR glasses
Manufacturing	Workbench	Assembly line	3D print
Payment	Cash	Credit card	Bitcoin
Photography	Analog camera	Digital camera	Drone camera
Communication	Dial telephone	Smartphone	Hologram phone
Housing	Hut	House	Smarthome
Time keeping	Sundial	Watch	Smartwatch
Weapon	Arc	Pistol	Lasergun

Pictures of each item (Experiments 1-3) can be found on the OSF (https://osf.io/25eb7/).

Table S2. Descriptive statistics for Experiment 1, related to Figure 1

Comparison	Innovativeness	IT		MT		AUC	
		M	*SD*	*M*	*SD*	*M*	*SD*
Past	Low	391.69	279.38	743.31	224.16	7,797.67	3,701.08
Past	High	394.04	288.31	755.13	226.30	9,030.86	3,727.21
Future	Low	397.48	297.51	826.61	237.36	9,543.35	3,729.70
Future	High	402.73	291.73	731.07	201.89	7,837.49	3,190.37

Table S2. Means and standard deviations of Initiation Time (IT), Movement Time (MT) and Area Under the Curve (AUC) for each combination of comparison type and innovativeness within Experiment 1.

Table S3. Descriptive statistics for Experiment 2, related to Figure 1

Comparison	Innovativeness	IT		MT		AUC	
		M	*SD*	*M*	*SD*	*M*	*SD*
Past	Low	363.10	250.23	786.81	257.73	8,987.95	3,139.58
Past	High	355.62	267.97	802.34	247.51	9,985.53	2,919.95
Future	Low	352.33	248.30	890.35	258.70	10,992.81	3,346.13
Future	High	350.97	222.97	775.59	265.51	8,641.65	2,895.06

Table S3. Means and standard deviations of Initiation Time (IT), Movement Time (MT) and Area Under the Curve (AUC) for each combination of comparison type and innovativeness within Experiment 2.

Table S4. Descriptive statistics for Experiment 3, related to Figure 1

Comparison	Innovativeness	IT		MT		AUC	
		M	*SD*	*M*	*SD*	*M*	*SD*
Past	Low	362.82	253.49	733.37	210.14	7,927.89	3,285.99
Past	High	350.52	238.07	785.59	255.06	9,205.41	3,718.09
Future	Low	358.27	266.63	862.30	260.36	10,260.75	4,051.81
Future	High	351.55	238.55	783.85	224.12	8,265.19	3,266.06

Table S4. Means and standard deviations of Initiation Time (IT), Movement Time (MT) and Area Under the Curve (AUC) for each combination of comparison type and innovativeness within Experiment 3.

Table S5. Descriptive statistics for Experiment 4, related to Figure 1

Comparison	Innovativeness	IT		MT		AUC	
		M	*SD*	*M*	*SD*	*M*	*SD*
Past	Low	297.92	219.62	909.67	288.42	9,843.53	3,258.52
Past	High	305.27	199.42	930.55	289.30	10,903.56	3,049.57
Future	Low	307.28	232.30	978.17	306.27	11,307.63	3,429.05
Future	High	299.32	222.88	845.58	256.72	9,362.61	3,072.15

Table S5. Means and standard deviations of Initiation Time (IT), Movement Time (MT) and Area Under the Curve (AUC) for each combination of comparison type and innovativeness within Experiment 4.

Data S1: Prestudy

To validate our item pool, we showed participants (n = 60 international adults) images of the respective objects in random order, and they had to rate their innovativeness and their typicality for a specific category (e.g., mobility). They indicated their response by moving a visual slider going from “Not innovative at all” to “Highly innovative” and from “Not typical at all” to “Highly typical”, respectively (responses were scaled from 0 to 100).

Within each category, the contemporary object (M = 55.08, SD = 21.56) was rated as more innovative than the historic one (M = 34.35, SD = 21.98), $t(59) = 10.55$, $p < .001$, d_z = 1.36, but as less innovative than the innovative option (M = 77.93, SD = 13.06), $t(59) = 8.80$, $p < .001$, d_z = 1.14. The contemporary option (M = 84.01, SD = 10.48) was rated as significantly more typical for the given category compared to the historic (M = 47.94, SD = 21.97), $t(59)$ = 13.97, $p < .001$, d_z = 1.80, and the innovative option (M = 43.49, SD = 22.05), $t(59) = 14.57$, $p < .001$, d_z = 1.88. The historic and the innovative option did not differ significantly in their degree of typicality, $t(59) = 1.91$, $p = .061$, d_z = 0.25. Table S1 shows the items we used within the experiments for each category.

Supplementary results

Interindividual differences

At the end of the study, we collected demographic information (self-reported age, gender and nationality). Furthermore, we included the *openness for experience scale* (Woo et al., 2014) and participants indicated their political orientation by moving a visual slider going from "very liberal" to "very conservative" (responses were scaled from 0 to 100).

As an exploratory analysis of interindividual differences regarding behavior in the main experiments, we calculated the difference between responses aiming at more and less innovative objects for each measure (IT, MT, AUC) and comparison type. This difference was correlated with participants' age, political orientation and the individual score obtained on the openness for experience scale. To account for multiple comparisons, the Holm-Bonferroni method was applied to correct the alpha level.

Experiment 1

For neither performance measure (IT, MT, AUC) or comparison type, we found a significant correlation of openness and the difference between traditional and innovative trials, $|r\text{s}| \leq .25$, $t\text{s} \leq 1.98$, $p\text{s} \geq .052$. Similar results were obtained for correlations with age, $|r\text{s}| \leq .15$, $t\text{s} \leq 1.20$, $p\text{s} \geq .236$, and political orientation, $t\text{s} < 1$.

Experiment 2

Also for Experiment 2, we found no correlation of the innovativeness effect with age, $|r\text{s}| \leq .22$, $t\text{s} \leq 1.66$, $p\text{s} \geq .103$, political orientation, $|r\text{s}| \leq .18$, $t\text{s} \leq 1.34$, $p\text{s} \geq .185$, or openness, $|r\text{s}| \leq .28$, $t\text{s} \leq 2.19$, $p\text{s} \geq .033$.

Experiment 3

In line with our findings for Experiment 1 and 2, there was no correlation of the innovativeness effect and age, $|r\text{s}| \leq .18$, $t\text{s} \leq 1.28$, $p\text{s} \geq .207$, political orientation, $|r\text{s}| \leq .22$, $t\text{s} \leq 1.62$, $p\text{s} \geq .112$, or openness, $|r\text{s}| \leq .28$, $t\text{s} \leq 2.04$, $p\text{s} \geq .046$, for Experiment 3.

Experiment 4

Also for Experiment 4 there was no correlation of the difference between more and less innovative trials and political orientation, $|r\text{s}| \leq .20$, $t\text{s} \leq 1.45$, $p\text{s} \geq .152$, age, $|r\text{s}| \leq .29$, $t\text{s} \leq 2.22$, $p\text{s} \geq .031$, or openness, $|r\text{s}| \leq .27$, $t\text{s} \leq 2.05$, $p\text{s} \geq .045$.
